# Social Participation of Marginalized Older Adults: Influence of Urban Context and Social Environment

**DOI:** 10.1093/geroni/igaf122.1717

**Published:** 2025-12-31

**Authors:** Mélanie Levasseur, Stéphanie Meynet, Mélissa Généreux, Shareck Martine, Sébastien Lord, Ruth Ndjaboue

**Affiliations:** Université de Sherbrooke, Sherbrooke, Quebec, Canada; Université de Sherbrooke, Sherbrooke, Quebec, Canada; Université de Sherbrooke, Sherbrooke, Quebec, Canada; Université de Sherbrooke, Sherbrooke, Quebec, Canada; Université de Montréal, Montréal, Quebec, Canada; Université de Sherbrooke, Sherbrooke, Quebec, Canada

## Abstract

To optimally promote health, it is essential to better consider the heterogeneity of older adults, especially in a context of a revitalization that can lead to various forms of gentrification and exacerbate the social exclusion of those at risk of marginalization. Using a participatory action research design, this study aimed to describe and explore the influence of three actions to foster the social participation of 20 older adults at risk of marginalization in a revitalizing urban city center. Guided tours of the local library were organized to share information about services and activities aimed at seniors and to help the library develop its tour offerings. Co-designed by, for, and with residents, 5 coffee meetings were held to provide older women living in a downtown low-income housing building with an opportunity to gather, access information, rebuild connections, and engage socially. Finally, in collaboration with the local fine arts museum, 4 artistic mediation workshops were implemented in the same building to increase participants’ social participation through various artistic techniques and to enable the museum to establish initial contact with a population that rarely attends its activities. The effects of these actions were explored through questionnaires and focus groups with older adults and stakeholders. The results highlight the importance of considering social dimensions during revitalization. Beyond physical improvements, it is crucial to integrate accessible and sustainable actions tailored to the abilities and needs of older adults at risk of marginalization, fostering a sense of community belonging and social participation.

